# Long Non-coding RNA LINC01119 Promotes Neuropathic Pain by Stabilizing BDNF Transcript

**DOI:** 10.3389/fnmol.2021.673669

**Published:** 2021-06-21

**Authors:** Le Zhang, Hao Feng, Yanwu Jin, Yufeng Zhan, Qi Han, Xin Zhao, Peilong Li

**Affiliations:** ^1^Department of Anesthesiology, The Second Hospital, Cheeloo College of Medicine, Shandong University, Jinan, China; ^2^Department of Anesthesiology, Affiliated Hangzhou First People’s Hospital, Zhejiang University School of Medicine, Hangzhou, China; ^3^Department of Clinical Laboratory, The Second Hospital, Cheeloo College of Medicine, Shandong University, Jinan, China

**Keywords:** neuropathic pain, LINC01119, BDNF, ELAVL1, spare nerve injury

## Abstract

Neuropathic pain (NP) is caused by primary injury or dysfunction of the peripheral and the central nervous system. Long non-coding RNAs were critical regulators involved in nervous system diseases, however, the precise regulatory mechanism remains unclear. This study aims to uncover the essential role of LINC01119 in NP progression and further clarify the underlying regulatory mechanism at post-transcriptional level. LINC01119 was significantly upregulated in rats of spare nerve injury (SNI) group compared to sham group. Functionally, silencing of LINC01119 significantly alleviated the neuropathic pain-induced hypersensitivity and reduced the increase in IL−6, IL−1β, and TNF−α caused by SNI. Mechanistically, Brain-derived neurotrophic factor (BDNF) was identified as the functional target of LINC01119. Besides, an RNA binding protein, ELAVL1 could directly interact with LINC01119, and this formed LINC01119- ELAVL1 complex binds to BDNF mRNA, strengthening its RNA stability and increasing the expression level of BDNF at both transcript and protein levels. Clinically, serum LINC01119 was verified as a promising diagnostic biomarker for NP patients. LINC01119 induces NP progression via binding with ELAVL1 and increasing BDNF mRNA stability and expression level. Therefore, LINC01119 may serve as a promising diagnostic marker and therapeutic target for NP treatment.

## Introduction

Neuropathic pain (NP) is a complicated, chronic pain state that is generally caused by tissue damage that affects the somatosensory nervous system (Nagpal et al., 2020). Neuropathic pain is linked with multiple diseases, commonly caused by Shingles (postherpetic neuralgia, PHN), diabetes (painful diabetic neuropathy, PDN) and others including trauma, stroke or cancer (Vuka et al., 2020). Pathologically, NP is tightly associated with peripheral and spinal cord injury and is a common clinical disease in orthopedics (Soler et al., 2021). However, the therapeutic approaches for NP are not satisfactory mainly due to the lack of effective molecular targets. Therefore, finding novel predictive markers and new targets to cure NP are urgently needed.

Long non-coding RNAs (lncRNAs) is characterized with the transcripts more than 200 nucleotides without protein coding potential (Serghiou et al., 2016). In recent years, lncRNAs were reported to play essential roles in various diseases, including cancer, cardiovascular, diabetes and neuropathy-related affaires; especially during the pathophysiological changes, such as cell growth and migration, autophagy and aging, et al. (Herrera-Solorio et al., 2017). Importantly, mountain evidence supports that a group of lncRNAs are involved in the central nervous system of rats and affects the development of nerves, and is closely related to many nervous system diseases (Pang et al., 2019; Song et al., 2020). However, the expression pattern, biological function, and underlying mechanism of lncRNAs in NP remains unclear.

RNA-binding proteins (RBPs) are playing wide roles in various diseases by the regulation of stability and splicing of mRNAs (Pereira et al., 2017). RNA-binding proteins regulate the processes of RNA maturation and decay to control the initiation and development of diseases (Upadhyay et al., 2013). For instance, ELAVL1 RBP (HuR) could stabilize matrix metalloproteinase-9 mRNA during seizure-induced mmp-9 expression in neurons, participating in the neuron lesions (Zybura-Broda et al., 2018). In addition, HuR was a critical regulator of autophagy during the process of myocardial ischemia-reperfusion injury, resulting in the progression of ferroptosis (Chen et al., 2021). Moreover, increasing studies suggested that lncRNAs could play essential roles in disease progression through the interaction with RBPs (Wu et al., 2016; Choi and Nam, 2018). However, whether lncRNAs promotes NP progression via binding with RBPs is not well known, and the related studies are very limited. Uncovering the regulatory mechanism of lncRNA-RBP interactions during NP progression could provide better understanding of NP treatment.

Brain-derived neurotrophic factor (BDNF) serves as a transducer, has been shown to contribute to the maintenance and development of NP by activating astrocytes and microglia as well as sensitizing neurons (Chow et al., 2020; Halloway et al., 2020). Previously, we identified the essential role of BDNF in NP progression (Zhang et al., 2016; Wang et al., 2017). However, how BDNF was regulated and the functional link between lncRNA and BDNF was not defined. In this study, we detected the expression of LINC01119 in NP model, and determined its role in NP progression. We revealed that LINC01119 was upregulated in rats with NP. Moreover, LINC01119 caused NP through upregulating BDNF expression via binding with ELVAL1.

## Materials and Methods

### Patient Samples

A total of 45 NP (postherpetic neuralgia, PHN) patients caused by Shingles and 49 healthy people were included from June 2018 to October 2020, to determine the predictive role of serum LINC01119. Serum samples were obtained and centrifuged at 3000 r/min for 10 min under 4°C. Included patients, aged from 24 to 78 years, had peripheral NP and a score ≥4/10 on pain VAS score (a 10-point VAS in which 0 indicated no pain and 10 indicated the worst possible pain). Evidences of neuropathy attributable to hypothyroidism, vitamin B12 deficiency, connective tissue disease, and amyloidosis were excluded. The study protocol was approved and written informed consent was obtained from all participants by the Research Scientific Ethics Committee of The Second Hospital of Shandong University.

### Animals

A total of 90 male Sprague-Dawley rats weighing 200–220 g were purchased from the Experimental Animal Center of Shandong University. Rats were housed in separated cages with a light/dark cycle of 12 h with free access to food and water. The room temperature was maintained at 26 ± 1°C, and the humidity was controlled at 40–50%. As previously described (Zhang et al., 2016), NP model in rats was constructed by performing SNI experiments. Rats were randomly divided into 9 groups: ① Sham (17 rats), ② Sham + +sh-LINC01119 (8 rats), ③ SNI (17 rats), ④ SNI-sh-NC (8 rats), ⑤ SNI-sh-LINC01119 (8 rats), ⑥ SNI-sh-LINC01119 + Lv-BDNF (8 rats), ⑦ SNI-Lv-NC (8 rats), ⑧ SNI-Lv-LINC01119 (8 rats), ⑨ SNI-Lv-LINC01119 + sh-ELAVL1 (8 rats). Among the above groups, five rats were used for evaluation of pain behaviors, and the rest were used for detection of LINC01119, BDNF, and ELAVL1 expression levels after behavioral test at day 14. The animal studies were carried out in accordance with NIH Guidelines for the Care and Use of Laboratory Animals and approved by the Animal Care Committee of the Second Hospital of Shandong University.

### Cell Culture

Rat microglial cells were purchased from Scien-cell Research Laboratories (Carlsbad, CA). Cells were cultured in Dulbecco’s Modified Eagle medium (DMEM; Lonza Inc. United States) supplemented with 10% FBS (Gibco, Carlsbad, CA) and 1% penicillin/streptomycin (Gibco) in an incubator at 37°C with humidified atmosphere of 5% CO_2_. Cells were authenticated by short tandem repeat (STR) profiling. Actinomycin D (ActD) was used for testing RNA stability and used for triplicates.

### Spare Nerve Injury (SNI) Surgery

Spare Nerve Injury models were established as previously described (Decosterd and Woolf, 2000). All rats were anesthetized with an intraperitoneal injection of 10% chloral hydrate (300 mg/kg). By incising the skin directly on the outside of the thigh and deep in the biceps femoris, the sciatic nerve and its three terminal branches were exposed. The tibia and common peroneal nerve were tightly ligated with 5–0 silk and cut 2–4 mm when present. The skin and muscle were sutured in two layers, and after this operation, the nerve remained completely flat and transparent. Sham-operated rats that underwent the same surgical procedure but nerves not damaged were used as a control group.

### Intrathecal Catheter Implantation and Lentivirus Injection

To stably overexpress or silencing LINC01119, BDNF, and ELAVL1, lentivirus-based vectors containing respective oligonucleotides were constructed by GenePharma (Shanghai, China). For cell transfection, the above-mentioned lentivirus vectors were mixed with 6 μg/ml polybrene and co-cultured for 24 h. For rat injection, we used an intrathecal catheter implantation method as previously described. Briefly, a PE-10 polyethylene catheter was inserted into the epidural space between the L5 and L6 vertebrae. After the anesthesia was completely restored, the 2% lidocaine (0.2 mL) was injected, and the dragging or paralysis of the hind limbs were observed to determine the correct implantation. The inside of the catheter is fixed with paraspinal muscles. After the wound is closed, the outside of the catheter is inserted and fixed on the skin and sutured to the head. The lentivirus vectors were injected via the above established intrathecal catheter. Brain-derived neurotrophic factor and ELAVL1 vectors were injected at day 3 while LINC01119-related vectors were injected at day 7 after SNI induction. The pain behaviors influenced by above injections were observed at day 14, 17, and 21 and repeated five times. Expression detection of respective genes were performed in day 14 and repeated in triplicates.

### NP Assessment

Mechanical sensitivity was assessed by determining paw withdrawal threshold (PWT). Rats were exposed in a transparent plastic box (22 × 12 × 22 cm) with a metal mesh at the bottom. Calibrated von Frey filament (IITC, Woodland Hills, CA, United States) was used to apply pressure on the plantar surface of the hind paw. The diameter of the filament is recorded when paw is pulled. Paw Withdrawal Latency (PWL) to noxious cold (0°C) was examined to evaluate cold allodynia. It was recorded by the length of time between the placement of the hind paw on the plate and a flinching of the paw. To avoid tissue damage, a cut off time of 40s for rats was used. Tests were performed 1 day before and 3, 7, 10, 14, 17, and 21 days after surgery, and all tests were repeated five times.

### RNA Extraction and qRT-PCR Analysis

Total RNAs was extracted by using L4-L5 spinal cord tissues or dorsal root ganglia (DRGs) or cells by using TRIzol reagent (Invitrogen, Carlsbad, CA) according to the manufacture’s guidelines. The quality and concentration of RNA were measured on a Nanodrop spectrophotometer (ND-1000, Nanodrop Technologies). cDNA was synthesized using Transcriptor First Strand cDNA Synthesis Kit (ROCHE, Basel, Switzerland) with corresponding specific primers. FastStart Essential DNA Green Master (ROCHE, Basel, Switzerland) was used for qRT-PCR on a LightCycler480 machine (ROCHE, Basel, Switzerland). GAPDH was used as the internal control to normalize lncRNA and mRNA expressions. The relative expression levels were determined by the 2^–ΔΔCt^ method and performed in triplicates. Related primers were detailed in Table 1.

**TABLE 1:** Information of the sequences of qPCR primer sequences.

**Primer name**	**Sequence (5’-3’)**
LINC01119 (Forward)	CCAGGCCCATCAATCACCTT
LINC01119 (Reverse)	GGCCTGTGTTCTGGCTACAT
BDNF (Forward)	GCCAGGGGCAACTCATCTTC
BDNF (Reverse)	GGTTGAAAGGCGCAGATGTC
ELAVL1 (Forward)	TTCCGCCATGTGGTCTTCAT
ELAVL1 (Reverse)	ATACACTGACTGTGGCAAGGT
GAPDH (Forward)	AGTTAATGCCGCCCCTTACC
GAPDH (Reverse)	CAGGGCTGACTACAAACCCA
U6 (Forward)	CTCGCTTCGGCAGCACA
U6 (Reverse)	AACGCTTCACGAATTTGCGT

### RNA Immunoprecipitation (RIP)

RNA Immunoprecipitation experiments were performed with a Magna RIP RNA-Binding Protein Immunoprecipitation Kit (Cat. 17-700) (Millipore, Cambridge, MA) following the manufacturer’s instructions. In brief, total RNA was extracted from L4 to L5 spinal cord tissues using TRIZOL reagent (Invitrogen) and then fragmented using 2μL fragmentation buffer later. The fragmented RNA was then incubated with anti-bodies against ELAVL1 (cat. no. ab200342, Abcam, Cambridge, MA) and IgG (cat. no. 12-371, EMD Millipore, Cambridge, MA) conjugated with A/G magnetic beads in IP buffer at 4°C for 2 h for immunoprecipitation. Next, the bound RNA was eluted from the beads in IP buffer. SuperScript^TM^ III Reverse Transcriptase (Invitrogen) was applicated for reverse transcription of the eluted RNA after purification. qRT-PCR was conducted using qPCR SYBR Green Master Mix (CloudSeq, Shanghai, China) in QuantStudio 5 real-time PCR System (Thermo Fisher, Waltham, MA, United States). The CT difference between input and the immunoprecipitated RNA was identified, and the relative enrichment was calculated using the 2 ^–ΔΔCt^ method. The experiments were repeated in triplicates.

### RNA Pulldown

LINC01119 was transcribed using T7 RNA polymerase or T3 RNA polymerase to obtain sense and antisense RNA. RNA Pull down assay for investigating RNA-protein interaction was performed by Pierce Magnetic RNA-Protein Pull-Down Kit (Thermo Fisher Scientific). Then the biotin-labeled RNA was mixed with 50 μl streptavidin magnetic beads 65801D (Invitrogen) which were pre-washed twice with Tris-Buffer. After incubation at room temperature for 30 min, the RNA-labeled beads were washed twice with tris-buffer. Then, 1 mg protein was added to the RNA-labeled beads in 1X Protein-RNA binding buffer. The mixture was incubated at 4°C with rotation overnight. Then the RNA-labeled beads with proteins were washed twice with wash buffer. RNA-interacting proteins were eluted with 50 μl elution buffer by incubation at 37°C for 30 min. Proteins were then run in an SDS-PAGE gels. Experiments were repeated in triplicates.

### RNA Fluorescent *in situ* Hybridization (RNA-FISH)

Oligonucleotide-modified probe sequence for LINC01119 was synthesized from GenePharma (Shanghai, China). Tissues were fixed by 4% paraformaldehyde followed by permeabilization with 0.5% Triton at room temperature for 15 min. The probe is pre-denatured at 73°C for 5 min. Then hybridization was performed with 2μM probe at 37°C overnight in a dark moist chamber. After being washed twice in 2 × SSC for 5 min, the slices were incubated with rabbit anti-BDNF antibody (Abcam, cat. no. ab108319). The images were acquired using a fluorescence microscopy ZFM-700 (Carl Zeiss AG, Oberkochen, Germany) rabbit anti-ELAVL1 antibody (1:1000, cat. no. ab200342, Abcam, Cambridge, MA). Experiments were repeated in triplicates.

### Immunofluorescence Staining Analysis

Immunofluorescence staining was performed on frozen coronal sections of L4–L5 rat spinal cords. For marker/BDNF or ELAVL1/BDNF double immunofluorescence, slides were incubated with a series of primary antibodies mixed with rabbit anti-BDNF antibody (1:500, cat. no. ab108319, Abcam, Cambridge, MA), and rabbit anti-ELAVL1 antibody (1:500, cat. no. ab200342, Abcam, Cambridge, MA), or anti-Iba1 antibody (1:500, cat. no. ab178846, Abcam, Cambridge, MA), or anti-NeuN (1:300, cat. no. ab177487, Abcam, Cambridge, MA) or anti-GFAP (1:500, cat. no. ab7260, Abcam, Cambridge, MA) overnight at 4°C. Then, tissues were incubated with same-origin secondary antibodies conjugated by FITC or GFP for 1 h at 37°C. Finally, the staining slides were pictured using a laser confocal microscope (TCS SP2 AOBS), and the related analysis were repeated in triplicates.

### Enzyme-Linked Immunosorbent Assay (ELISA)

Enzyme-linked immunosorbent assay (R&D System, United States) was performed according to the instructions to measure the protein expression level of IL-6, TNF-α, and IL-1β in the L4–L5 rat spinal cord tissue (obtained 14 days after the establish of SNI model). Tissue samples were centrifuged for 10 min at 10000 rpm, and the supernatants were collected and stored at -80°C for future analysis. The experimental steps were strictly performed according to the manufacturer’s manual and repeated in triplicates.

### Western Blot Analysis

Western blot was performed to measure protein expression levels in L4–L5 rat spinal cords and microglial cells. The following primary antibodies were used: rabbit anti-BDNF antibody (1:1000, cat. no. ab108319, Abcam, Cambridge, MA), rabbit anti-ELAVL1 antibody (1:1000, cat. no. ab200342, Abcam, Cambridge, MA), rabbit anti-α-Tubulin (1:1000, cat. no. ab7291, Abcam, Cambridge, MA), rabbit anti-GAPDH (1:1000, cat. no. ab8245, Abcam, Cambridge, MA), secondary antibody goat anti-rabbit IgG (1:5000, cat. no. ab6721, Abcam, Cambridge, MA). Proteins were extracted using RIPA buffer (Beyotime, Shanghai, China). Protein extracts were separated with 10% SDS-PAGE, and then electro-transferred to polyvinylidene difluoride membrane. The membrane was then blocked with 5% fat-free milk followed by incubation with respective primary antibodies at 4°C overnight. The detection of proteins was carried out using ECL reagent. Experiments were repeated in triplicates.

### Statistical Analysis

Statistics were presented as mean ± SD. Comparisons between two groups were carried out via paired or unpaired Student’s *t*-test accordingly. Comparisons among multiple groups were performed using one-way analysis of variance with Tuker-*post hoc* test. Fisher exact testing was performed to evaluate the difference of proportions between different groups. Statistical analyses were performed using GraphPad Prism (v8.0.1, GraphPad Software Inc., San Diego, CA, United States). *P* < 0.05 was used for the indication of statistical significance.

## Results

### LINC01119 Is Upregulated in the Spinal Cord of SNI-NP Rat Model

**FIGURE 1:**
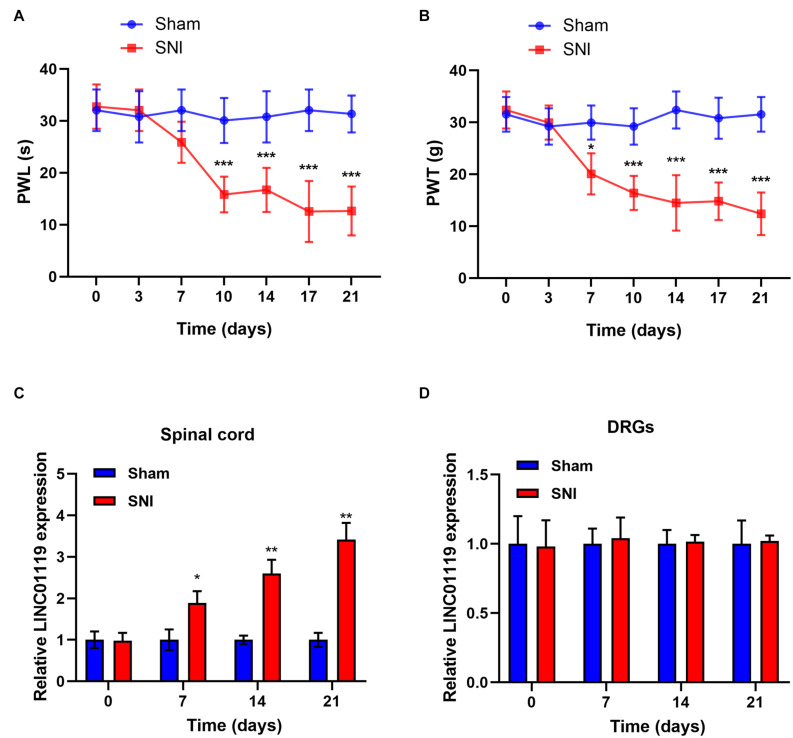
LINC01119 was upregulated in rats with SNI **(A,B)** PWL **(A)** and PWT **(B)** in rats of Sham group and SNI group, *P < 0.05, ***P < 0.001 compared to Sham group, *n* = 5/group. **(C,D)** The expression level of LINC01119 were detected in rats of SNI and control group from spinal cord **(C)** and DRGs **(D)** at day 0, 7, 14, and 21. *P < 0.05, **P < 0.01 compared to Sham group, *n* = 3/group.

Firstly, we established NP rats by using SNI model. No significant difference in PWL and PWT between groups prior to the operation was identified. In response to the operation, PWL and PWT were significantly decreased in SNI rats while no significant change was identified in sham rats (Figures 1A,B). Previously, it has been reported that LINC01119 may be bio-molecules that are closely related to NP (Zhao et al., 2021). To validate the expression mode of LINC01119 in NP rats, we performed qRT-PCR using L4–L5 spinal cord and DRGs samples from SNI rats and sham rats. Figure 1C showed that LINC01119 expression was significantly higher in the spinal cord tissues of NP rats compared to controls. However, LINC01119 was unchanged in DRGs of SNI rats (Figure 1D), suggesting that LINC01119 may participated in NP in spinal cord areas.

### Silencing of LINC01119 Attenuates NP at Spinal Cord of Rats Undergoing SNI

**FIGURE 2:**
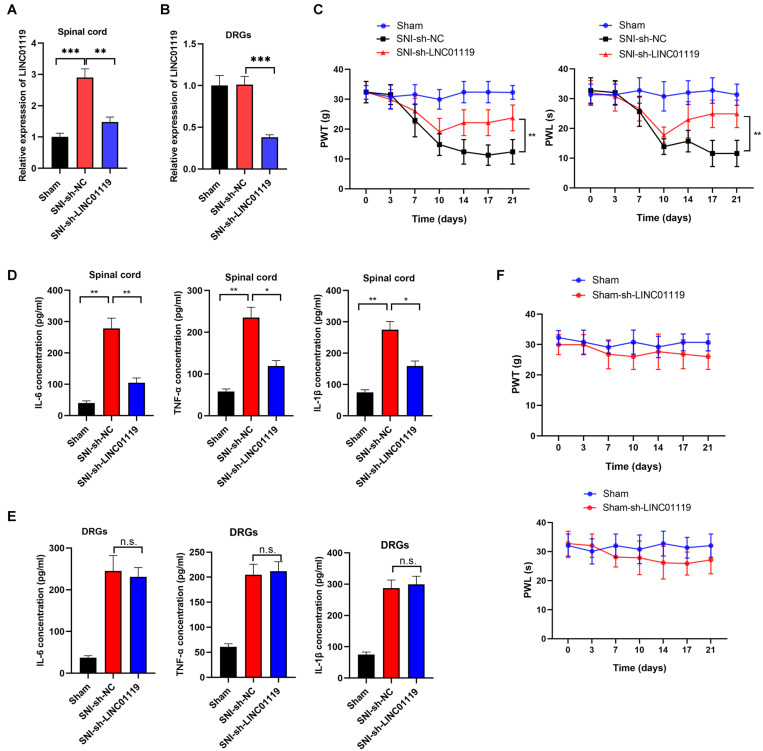
LINC01119 causes NP in rats undergoing SNI **(A,B)**. LINC01119 was silenced in SNI rats after intrathecal injection of sh-LINC01119 vector in both spinal cord **(A)** and DRGs **(B)**, **P < 0.01, ***P < 0.001, *n* = 3/group. **(C)** PWT (left) and PWL (right) were assessed in rats silenced with LINC01119, **P < 0.01, *n* = 5/group. **(D,E)** The concentrations of IL–6, TNF–α, and IL-1β in spinal cord **(D)** and DRGs tissues **(E)** of rats were detected by ELISA at day 14, *P < 0.05, **P < 0.01, *n* = 3/group. **(F)** PWL and PWT were not altered by LINC01119 knockdown in sham rats without SNI surgery, *n* = 5/group.

To verify the functional role of LINC01119 in NP, LINC01119-silencing vector (sh-LINC01119) and a negative control vector (sh-NC) were injected intrathecally at day 7 after SNI induction. The expression of LINC01119 at day 14 was significantly decreased after intrathecal administration of sh-LINC01119 in both spinal cord and DRGs tissues (Figures 2A,B). In addition, silencing of LINC01119 significantly attenuated NP behaviors as evidenced with restored PWL and PWT (Figure 2C). It is well known that inflammation-associated cytokines, including IL-6, TNF-α, and IL-1β, were closely associated with neuropathic pain, hence we determined their expression levels via ELISA at day 14 in SNI rats after intrathecal injection of sh-LINC01119 at day 7. Results showed that the concentrations of IL-6, TNF-α, and IL-1β were upregulated in SNI rats and significantly reversed by the inhibition of LINC01119 in spinal cord tissues (Figure 2D), however, this inhibition of inflammatory cytokines by LINC01119 was not observed in DRGs (Figure 2E), suggesting that LINC01119 may regulate neuroinflammation in spinal cord area but not in DRGs. Moreover, this functional of LINC01119 in NP was not observed in sham rats without SNI surgery (Figure 2F). Therefore, the above data suggested that LINC01119 play critical roles in neuroinflammation and NP behaviors in spinal cord area of SNI rats.

### BDNF Mediates the LINC01119-Induced NP in Spinal Cord of SNI Rats

**FIGURE 3:**
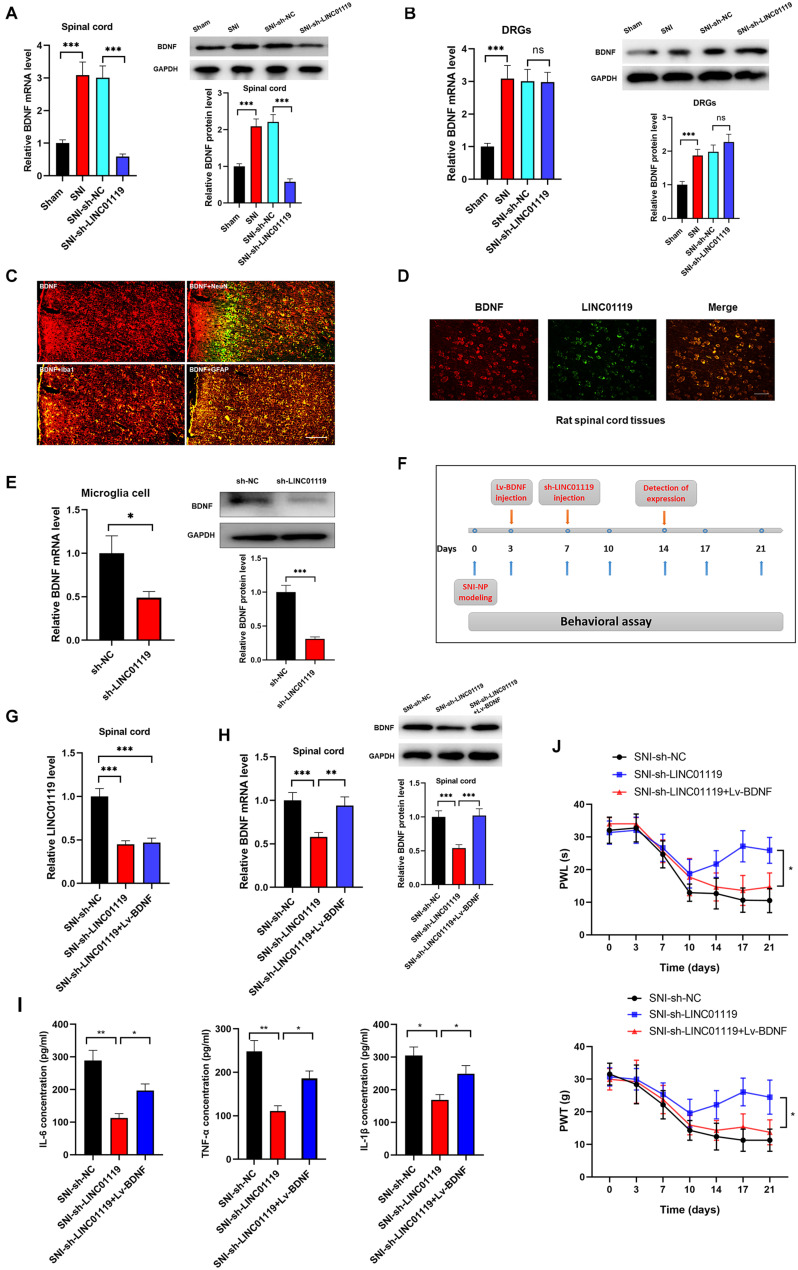
LINC01119 mediated NP progression via targeting BDNF. **(A)** BDNF mRNA (left panel) and protein (right panel) were upregulated in spinal cord tissue of SNI rats and downregulated by intrathecal injection of sh-LINC01119 in contrast to sham rats, ***P < 0.001, *n* = 3/group. **(B)** BDNF mRNA (left panel) and protein (right panel) were upregulated in DRGs of SNI rats, but was not regulated by sh-LINC01119, ***P < 0.001, *n* = 3/group. **(C)** Localization of BDNF in the spinal cord of SNI rats by double immunofluorescence of BDNF (red) with NeuN (marker of neuron, merged as yellow), lba1 (marker of microglia, merged as yellow), and GFAP (marker of astrocyte, merged as yellow). Scare bar = 100μm. **(D)** FISH analysis revealed a co-localization of LINC01119 and BDNF in spinal cord. Scare bar = 100μm. **(E)** BDNF was downregulated in microglia cells silenced with LINC01119 at both transcript and protein levels compared to control group, *P < 0.05, ***P < 0.001, *n* = 3/group. **(F)** The procedure of intrathecal injection of respective lentivirus vectors. **(G,H)** Expressions of LINC01119 **(G)** and BDNF **(H)** were shown in respective groups with different manipulations, **P < 0.01, ***P < 0.001, *n* = 3/group. **(I)** The concentrations of IL–6, TNF–α, and IL-1β in the spinal cord tissue of rats were downregulated by LINC01119 knockdown, however, this effect was abrogated by overexpression of BDNF, *P < 0.05, **P < 0.01, *n* = 3/group. **(J)** PWL and PWT was evaluated in SNI rats which were silenced of LINC01119 with or without BDNF overexpression, *P < 0.05, *n* = 5/group.

Brain-derived neurotrophic factor (BDNF) has been well accepted as an activity-dependent neuronal regulator in the nervous system via enhancing neuronal excitability. Previously, we demonstrated that BDNF contributes to neuropathic spontaneous pain in SNI rats (Zhang et al., 2016; Wang et al., 2017). In this study, we sought to define whether LINC01119 regulates NP progression via modulating BDNF expression. Brain-derived neurotrophic factor was downregulated in spinal cord tissues, but was not altered in DRGs after injection of sh-LINC01119 (Figures 3A,B), suggesting that LINC01119 may influence NP via regulating BDNF expression in spinal cord areas. We then detected BDNF distribution via performing double immunofluorescence assay. Figure 3C showed that BDNF was co-localized with NeuN (a neuronal marker), Ibal (a microglial marker), and GFAP (an astrocytic marker), indicating that BDNF was diffusely located in both glial cells and neurons of spinal cord. With specific LINC01119 probe, we also verified the co-localization of LINC01119 and BDNF (Figure 3D). In addition, BDNF was also suppressed by silencing of LINC01119 in rat microglia cells (Figure 3E). Subsequently, we overexpressed BDNF in LINC01119-silenced SNI rats by intrathecal injection of Lv-BDNF (Figure 3F). LINC01119 and BDNF expression in spinal cord was successfully manipulated at day 14 in respective groups (Figures 3G,H). Analysis with ELISA showed that BDNF overexpression could partially attenuate the suppressive effect of sh-LINC01119 on the expressions of IL-6, TNF-α, and IL-1β in SNI rats (Figure 3I). Consistently, the effects on NP-like behaviors caused by knockdown of LINC01119 were also abrogated by overexpression of BDNF (Figure 3J). Above results clearly showed that BDNF was essential for LINC01119-induced NP in spinal cord of SNI rats.

### LINC01119 Directly Interacts With ELAVL1

**FIGURE 4:**
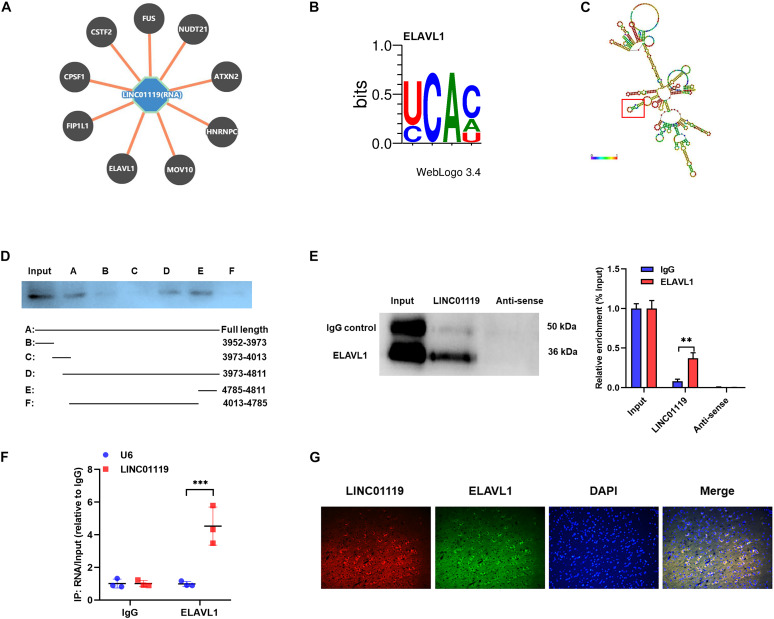
LINC01119 directly interacts with ELAVL1. **(A)** The potential binding proteins of LINC01119 predicted by POSTAR (http://lulab.life.tsinghua.edu.cn/postar/index.php). **(B)** Predicted motif sequence of ELAVL1 was shown according to POSTAR. **(C)** The region of chr2:46834785-46834811 formed a stem-loop structure as predicted according to RNAfold (http://rna.tbi.univie.ac.at//cgi-bin/RNAWebSuite/RNAfold.cgi?). **(D)** Serial deletion analysis revealed that the chr2:46834785-46834811 region of LINC01119 was essential for binding with ELAVL1. **(E)** RNA pulldown assay using biotinylated *LINC01119* and antisense control were performed using spinal cord of SNI rats, **P < 0.01, *n* = 3/group. **(F)** RIP assay with ELAVL1 antibody further confirmed the direct interaction between LINC01119 and ELAVL1 in SNI rats, ***P < 0.001, *n* = 3/group. **(G)** RNA-FISH with specific probe using SNI rats verified that LINC01119 and ELAVL1 protein colocalized mostly in the cytoplasm section (*n* = 3/group). Scare bar = 100μm.

To verify the RBPs of LINC01119, we performed bioinformatic analysis with *POSTAR*^1^ and revealed the interacting networks (Figure 4A). Six binding sites of embryonic lethal abnormal version like RNA binding protein 1 (ELAVL1) were predicted at the function region (chr2:46833952-46834811) of LINC01119 (Table 2, Figure 4B). Then, we analyzed the structure of binding area of LINC01119 on *RNAfold* online software (Lorenz et al., 2011), and revealed that the targeted area could form a stem-loop, which is essential for the binding with RBPs (Figure 4C). Furthermore, by performing serial deletion analysis, we verified that the region of chr2:46834785-46834811 of LINC01119 was essential for binding with ELAVL1 (Figure 4D). Take a step further, we verified that ELAVL1 could directly bind with LINC01119 in spinal cord tissues by performing western blotting after RNA pulldown (Figure 4E). RIP assay also proved the direct binding between ELAVL1 with LINC01119 (Figure 4F). RNA Fluorescent *in situ* Hybridization using spinal cord tissues showed that LINC01119 and ELAVL1 were co-expressed, which strengthens our conclusion (Figure 4G). Collectively, our results clearly showed that LINC01119 could directly interact with ELAVL1, suggesting a potential regulatory role of LINC01119-ELAVL1 complex in NP progression in SNI rats.

**TABLE 2:** Predicted targeted sites of LINC01119 by ELAVL1 in *POSTAR.*

**Targeted site**	**Location**	**Score**	**PhastCons**	**Phylop**
1	chr2:46833952-46833973	0.703464	0.0638095	0.219905
2	chr2:46833952-46833973	0.703464	0.0638095	0.219905
3	chr2:46833987-46834013	0.800532	0.430038	0.337692
4	chr2:46833987-46834013	0.800532	0.430038	0.337692
5	chr2:46834785-46834811	0.742447	0.00226923	–0.0092692
6	chr2:46834785-46834811	0.742447	0.00226923	–0.0092692

### LINC01119-ELAVL1 Complex Stabilizes BDNF mRNA, Thereby Inducing NP in SNI Rats

**FIGURE 5:**
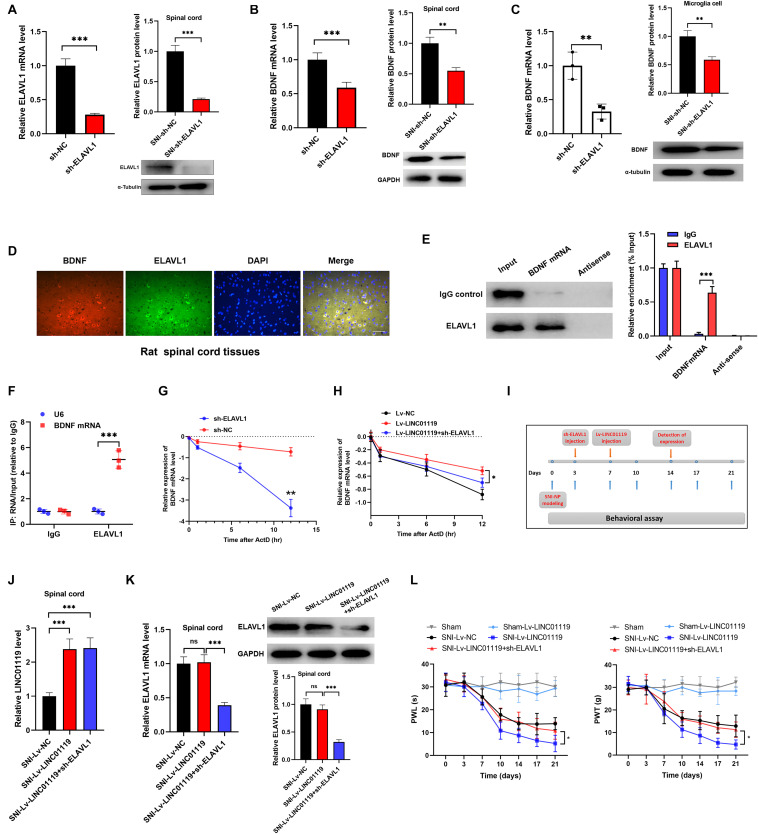
LINC01119-ELAVL1 complex stabilizes BDNF mRNA, thereby inducing NP in SNI rats. **(A)** ELAVL1 was silenced in spinal cord tissue of SNI rats at both transcript and protein levels, ***P < 0.001, *n* = 8/group. **(B)** BDNF was downregulated in spinal cord tissue of SNI rats silenced with ELAVL1 at both transcript and protein levels, ***P < 0.001, *n* = 3/group. **(C)** Knockdown of ELAVL1 induced inhibition of BDNF expression in rat microglial cells, **P < 0.01. **(D)** Immunofluorescence showed the co-expression of ELAVL1 and BDNF protein in spinal cord tissue of SNI rats. Scare bar = 100μm. **(E)** RNA pulldown assay showed that ELAVL1 protein was interacted with BDNF mRNA in spinal cord tissues, ***P < 0.001, *n* = 3/group. **(F)** RIP assay with ELAVL1 antibody in spinal cord showed a significantly higher enrichment of BDNF mRNA compared to U6, ***P < 0.001, *n* = 3/group. **(G)** The half-life of BDNF RNA was dramatically decreased in ELAVL1-silenced cells compared to that in control cells, ***P < 0.001. **(H)** Knockdown of ELAVL1 reversed the LINC01119-mediated RNA stability of BDNF in microglia cells, *P < 0.05. **(I)** The flow chart of manipulations of respective lentivirus vectors in SNI rats. **(J,K)** Expressions of LINC01119 **(J)** and ELAVL1 **(K)** in groups received respective manipulations, ***P < 0.001, *n* = 3/group. **(L)** PWL and PWT assays showed that knockdown of ELAVL1 abrogated LINC01119-induced NP behaviors, *P < 0.05, *n* = 5/group.

Previous literatures demonstrated that ELAVL1 was a critical regulator in stabilizing mRNAs by linking with AU-rich elements (AREs) (Bakheet et al., 2018). To confirm the LINC01119-ELAVL1 complex could increase BDNF level via stabilizing BDNF transcript, we knocked out ELAVL1 by intrathecally injection of sh-ELAVL1 (Figure 5A). As shown in Figure 5B, downregulation of ELAVL1 induced decreased expression of BDNF in spinal cord at both transcript and protein levels, suggesting that ELAVL1 may function synchronously with the interaction of LINC01119. In rat microglial cells, silencing of ELAVL1 also induced decreased expression of BDNF at both transcript and protein level (Figure 5C). By performing immunofluorescence assay, we observed a co-expression of ELAVL1 and BDNF (Figure 5D) in spinal cord. RNA pulldown and RIP assay with ELAVL1 antibody provided direct evidence supporting the interaction between ELAVL1 and BDNF mRNA in NP rats (Figures 5E,F).

To verify the role of ELAVL1 in the regulation of BDNF stability, we blocked RNA transcription process by treating with actinomycin D (ActD) in rat microglial cells, the stability of BDNF RNA was dramatically suppressed by the silencing of ELAVL1 (Figure 5G). Moreover, enhanced expression of LINC01119 prevented the decay process of BDNF mRNA, however, this effect was significantly abrogated when ELAVL1 was knocked out, suggesting that ELAVL1 is essential for LINC01119-induced BDNF mRNA stability (Figure 5H). To demonstrate whether silenced ELAVL1 reverses LINC01119-mediated NP production, Lv-LINC01119 and sh-ELAVL1 vectors were injected intrathecally into SNI rats at day 7 and day 3, respectively (Figure 5I). The manipulations of LINC01119 and ELAVL1 were confirmed (Figures 5J,K). The results showed that silencing ELAVL1 abrogated the LINC01119-mediated NP behaviors (Figure 5L). Taken together, the above data suggests that LINC01119-ELAVL1 complex may directly bind to BDNF mRNA and regulate its stability, thereby causing NP progression.

### LINC01119 Is a Useful Biomarker for NP Diagnosis and Prognosis

**FIGURE 6:**
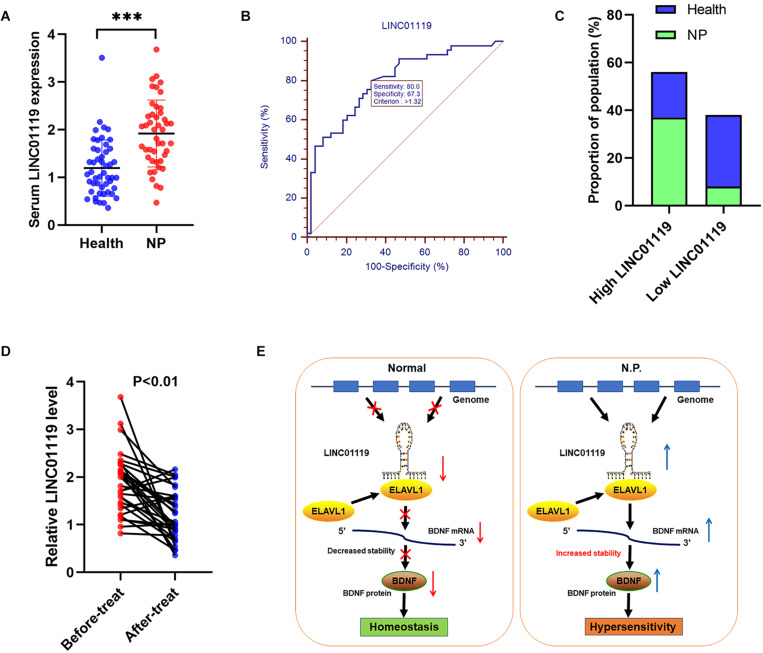
LINC01119 is a promising diagnostic marker for NP patients. **(A)** LINC01119 expression was detected in serum samples from 45 NP patients (PHN) and 49 healthy people by qRT-PCR, ^∗∗∗^*P* < 0.001. **(B)** ROC curve was established to evaluate the diagnostic performance of serum LINC01119 level. **(C)** The proportions of NP patients and healthy people in high or low-LINC01119 groups were shown. **(D)** Serum LINC01119 level were determined in 45 NP patients before and after treatment. **(E)** Schematic diagram of LINC01119 induces NP through regulating BDNF expression in an ELAVL1-dependent manner.

We also explored the clinical role of LINC01119 in patients with Shingles-induced NP; the detailed patient information showed that no statistical bias was identified between NP patients and controlled people (Table 3). By detecting the expression level of LINC01119 in serum samples, we verified that LINC01119 was significantly upregulated in NP patients compared to healthy individuals (Figure 6A). Receiver operator characteristic (ROC) analysis indicated a high diagnostic potential of serum LINC01119 in differentiating NP and healthy population, with sensitivity, specificity and AUC reaching 80.0%, 67.3%, and 0.799, respectively (Figure 6B). By stratifying all populations into a high and a low LINC01119-level group based on the stratification criterion of 1.32 according to the ROC curve, we revealed that the proportion of NP patients was much higher in high LINC01119-level group when compared to that of low LINC01119 group (Figure 6C), which further indicates LINC01119 is a promising diagnostic marker. We then defined the expression level of LINC01119 in NP patients in remission after treatment, and found that LINC01119 was dramatically downregulated in contrast to the level before treatment (Figure 6D). Therefore, our study provided a preliminary data showing the potential value of circulating LINC01119 in diagnostic and efficacy monitoring of NP patients.

**TABLE 3:** Characteristics of patients with NP and the healthy control group (CG).

**Characteristic**	**NP (*N* = 45)**	**CG (*N* = 49)**	**Statistical significance**
Age (y; mean ± SD)	57.4 ± 12.4	56 ± 12.8	*P* > 0.05
Gender (n; female/male)	26/19	29/20	*P* > 0.05
BMI (kg/m^2^; mean ± SD)	23.1 ± 3.1	22.6 ± 3.6	*P* > 0.05
HZ Location (n/N)			
•Thorax	21/45	-	-
•Abdomen	9/45	-	-
•Extremities	7/45	-	-
•Other	8/45	-	-
Pain VAS (0–10; mean ± SD)	6.0 ± 1.7	-	-

## Discussion

Despite current therapeutic regimens that evidently improved the prognosis of patients experienced nerve injury, nearly all those patients finally develop pain, which indirectly influence the quality of life (Fink et al., 2017; Nickel et al., 2001). Long non-coding RNAs are dysregulated in various diseases, and mediate biological process by interacting with RNA and proteins (Huang et al., 2018). However, the detailed regulatory pathways that involved in lncRNA-mediated NP progression is still not clearly defined. In this work, we explored the potential role of LINC01119 in NP via the post-transcriptional regulation of BDNF expression. We proved that LINC01119 accelerated NP formation via increasing BDNF expression. Mechanistically, LINC01119 directly interacted with ELAVL1 and the LINC01119-ELAVL1 complex could bind to and stabilize BDNF mRNA (Figure 6E).

NP remains a clinically challenging problem and the mechanisms are still unclear. The rat model of NP provides an appropriate system for preclinical study of pain (Kc et al., 2020). Previously, Zhao et al. compares lncRNA and mRNA expression and screens disease-related biomarkers related to NP after spinal cord injury (SCI) in peripheral blood samples of patients (Zhao et al., 2021). They revealed two lncRNAs, LINC01119, and LINC02447, are directly involved in the pain pathway and may be important for NP. In this study, we focused on those lncRNAs and eventually identified LINC01119 as a critical regulator of NP. In addition, LINC01119 was reported as a potential regulatory gene involved in cervical cancer (Ding et al., 2020), colorectal cancer (Han et al., 2019), and adipocyte differentiation (Chen et al., 2019). However, its detailed functional role and regulatory mechanism in NP and other diseases are still not well known. Our study determined that LINC01119 was upregulated in NP rat model in spinal cord area but not in DRGs. Furthermore, knockdown of LINC01119 significantly decreased the expression of IL-6, TNF-α, and IL-1β and partly reversed NP, suggesting an essential role of LINC01119 in the formation of NP in spinal cord. To the best of our knowledge, this is the first study to identify the functional role of LINC01119 in NP.

Another finding of this study is the identification of the RBP, ELAVL1 (also known as Hu Antigen R, HuR), which could directly bind to LINC01119. ELAVL1 is a ubiquitously expressed member of the embryonic lethal abnormal vision (ELAV)-like/Hu-protein family of RNA-binding proteins (Zhang et al., 2017). ELAVL1 selectively binds to target mRNAs bearing specific sequence elements, often U- and AU-rich, and generally found in the mRNA 3’-UTR, and plays a critical role in their posttranscriptional regulation (Chang and Hla, 2014). Multiple studies have demonstrated the essential role of ELAVL1 in the development of biological progression via the modulation at post-transcriptional mode, such as cancer metastasis, autophagy and others (Zhang et al., 2018; Palomo-Irigoyen et al., 2020). More importantly, Li et al. reported that ELAVL1 was involved in the lncRNA MALAT1-regulated renal tubular epithelial pyroptosis in diabetic nephropathy, revealing its potential role NP (Li et al., 2017). Our results suggest that there exists a direct interaction between LINC01119 and ELAVL1 protein, and the formed LINC01119-ELAVL1 complex further binds to BDNF mRNA, inducing stabilization of BDNF and increased expression. Reciprocally, overexpression of ELAVL1 partly reversed the LINC01119-caused NP effect, indicating that LINC01119 functions dependent on the interaction with ELAVL1. Our results also suggest that both LINC01119 and BDNF mRNA were interacted by ELAVL1 protein, however, the direct interaction between LINC01119 and BDNF mRNA were not observed, suggesting that ELAVL1 protein may function as a scaffold to form the LINC01119-ELAVL1-BDNF complex.

Brain-derived neurotrophic factor (BDNF) is a well-studied growth factor that serves many critical functions within the central nervous system (Almeida et al., 2021). It is a member of a unique family of polypeptide growth factors, neurotrophies, which influence the proliferation, differentiation, survival, and death of neuronal and non-neuronal cells (Cappoli et al., 2020; Su et al., 2020). Despite this, BDNF has been implicated in several injury-induced maladaptive processes including pain, spasticity and convulsive activity (Smith, 2014). Our previous study has well addressed the role of BDNF in neuropathic spontaneous pain-related aversion via NR2B receptors (Zhang et al., 2016). Other studies also reported the contribution of BDNF in NP (Fiore and Austin, 2019). This study further revealed how BDNF was regulated during NP and the direct association between LINC01119-ELAVL1 complex. Gain- and -loss functional assays showed that BDNF was essential for LINC01119-mediated NP. Moreover, dysregulation of BDNF expression could only partially reversed the LINC01119-induced NP and neuroinflammation, suggesting that there are other pathways targeted by LINC01119 involved in NP and neuroinflammation.

Finally, we also investigated the clinical role of LINC01119 as crucial biomarker used for NP diagnosis and curative effect monitoring. Currently, the clinical tools used for diagnosing NP are very limited (Jonsson et al., 2021). Finding effective clinical biomarkers for NP is an urgent issue. By detecting the serum LINC01119 level in NP patients, we revealed its clinical significance, suggesting that LINC01119 is a promising biomarker for the diagnosis and prognosis of NP. We need to point out the limitations of this study. First, experimental and preliminary clinical role of LINC01119 was identified in NP, however, a large cohort multicenter study was needed before clinical application. Second, although the functional role of LINC01119 and the underlying regulation pathway of LINC01119/ELAVL1/BDNF were identified, the detailed elements that participated in the initiation and progression of NP, such as other pain-related factors in the microenvironment involved in signaling transmission need to be defined in following studies.

## Conclusion

To conclude, our study revealed the novel contribution of LINC01119/ELALV1/BDNF axis in NP progression. Our discovery not only help us get a better understanding on the regulatory potential of LINC01119 in NP, but also useful for finding promising drug targets and developing novel therapeutic strategies to overcome NP.

## Data Availability Statement

The original contributions presented in the study are included in the article/Supplementary Material, further inquiries can be directed to the corresponding authors.

## Ethics Statement

The studies involving human participants were reviewed and approved by the Research Scientific Ethics Committee of The Second Hospital of Shandong University. The patients/participants provided their written informed consent to participate in this study. The animal study was reviewed and approved by the Animal Use and Care Committee of Shandong University.

## Author Contributions

LZ, HF, and PL acquired date and draft of the manuscript. LZ, YJ, and XZ established NP models and performed the experimental assays. LZ and YZ analyzed and interpreted date and statistical analysis. QH and YZ provide technical and material support. XZ and PL approved the final version of the manuscript. All authors contributed to the article and approved the submitted version.

## Conflict of Interest

The authors declare that the research was conducted in the absence of any commercial or financial relationships that could be construed as a potential conflict of interest.

## Footnotes

1 http://lulab.life.tsinghua.edu.cn/postar/index.php

## Abbreviations

AREs: AU-rich elements
BDNF: Brain-derived neurotrophic factor
ELAVL1: embryonic lethal abnormal version like RNA binding protein 1
ELISA: Enzyme-linked immunosorbent assay
IHC: Immunohistochemistry
lncRNAs: Long non-coding RNAs
mRNA: messenger RNA
NP: Neuropathic pain
PWT: Paw withdrawal threshold
PWL: paw withdrawal latency
RBPs: RNA-binding proteins
RIP: RNA immunoprecipitation
RNA-FISH: RNA fluorescent *in situ* hybridization
ROC: Receiver operator characteristic
SCI: spinal cord injury
SNI: spare nerve injury.
